# Investigating Non-Native Ribbon Worm *Cephalothrix simula* as a Potential Source of Tetrodotoxin in British Bivalve Shellfish

**DOI:** 10.3390/md22100458

**Published:** 2024-10-05

**Authors:** Monika Dhanji-Rapkova, Robert G. Hatfield, David I. Walker, Chantelle Hooper, Sarah Alewijnse, Craig Baker-Austin, Andrew D. Turner, Jennifer M. Ritchie

**Affiliations:** 1 Centre for Environment, Fisheries and Aquaculture Science (Cefas), Barrack Road, Weymouth DT4 8UB, UK; robert.hatfield@cefas.gov.uk (R.G.H.); david.walker@cefas.gov.uk (D.I.W.); chantelle.hooper@cefas.gov.uk (C.H.); sarah.alewijnse@cefas.gov.uk (S.A.); craig.baker-austin@cefas.gov.uk (C.B.-A.); andrew.turner@cefas.gov.uk (A.D.T.); 2 Faculty of Health and Medical Sciences, University of Surrey, Guildford GU2 7XH, UK; j.ritchie@surrey.ac.uk

**Keywords:** tetrodotoxin, *Cephalothrix simula*, bivalve shellfish, Pacific oysters, Great Britain

## Abstract

Tetrodotoxin (TTX) is a potent marine neurotoxin found in several phylogenetically diverse organisms, some of which are sought as seafood. Since 2015, TTX has been reported in bivalve shellfish from several estuarine locations along the Mediterranean and European Atlantic coasts, posing an emerging food safety concern. Although reports on spatial and temporal distribution have increased in recent years, processes leading to TTX accumulation in European bivalves are yet to be described. Here, we explored the hypothesis that the ribbon worm species *Cephalothrix simula*, known to contain high levels of TTX, could play a role in the trophic transfer of the toxin into shellfish. During a field study at a single location in southern England, we confirmed *C. simula* DNA in seawater adjacent to trestle-farmed Pacific oysters *Magallana gigas* (formerly *Crassostrea gigas*) with a history of TTX occurrence. *C. simula* DNA in seawater was significantly higher in June and July during the active phase of toxin accumulation compared to periods of either no or continually decreasing TTX concentrations in *M. gigas*. In addition, *C. simula* DNA was detected in oyster digestive glands collected on 15 June 2021, the day with the highest recorded *C. simula* DNA abundance in seawater. These findings show evidence of a relationship between *C. simula* and TTX occurrence, providing support for the hypothesis that bivalves may acquire TTX through filter-feeding on microscopic life forms of *C. simula* present in the water column at particular periods each year. Although further evidence is needed to confirm such feeding activity, this study significantly contributes to discussions about the biological source of TTX in European bivalve shellfish.

## 1. Introduction

Tetrodotoxin (TTX) is a heat-stable neurotoxin commonly associated with the pufferfish species (*Tetraodontidae* family), which have been responsible for nearly 60% of TTX-poisoning cases reported, predominantly in Southeast and East Asia [1]. TTX can accumulate in other edible marine organisms, such as arthropods (*Carcinoscorpius rotundicauda*) and molluscs (mainly the gastropod family *Nassaridae*), including bivalve molluscs (reviewed in [2,3,4,5,6,7,8]). The first incidence of TTX in bivalve shellfish was reported in Japan in 1993 [9], followed by New Zealand in 2012 [10]. However, TTX in European bivalves was not documented until 2015 [11,12]. Since then, TTX screening studies varying in scale have been conducted in several countries along the Mediterranean and Atlantic coasts of Greece, Italy, France, Portugal, Spain, the Netherlands, and the United Kingdom (UK) [13,14,15,16,17,18,19,20,21,22,23,24,25].

While information about the spatial and temporal distribution of TTX in bivalve shellfish has expanded, little is known about the processes leading to TTX accumulation. A commonly accepted hypothesis considers marine bacteria as TTX producers (reviewed in [26]). A trophic transfer involving phytoplankton has been suggested [9,12], supported by findings of higher TTX concentrations in digestive glands compared to other tissues in some bivalves [13,27,28,29]. Accumulation of marine biotoxins in shellfish through filter-feeding on toxin-producing microalgae is a well-known phenomenon and a food safety threat [30,31,32]. In this context, several dinoflagellate species have been investigated as potential TTX sources, although conclusive evidence of their role in TTX production or transfer is still lacking [9,12,13,15,20,21,25,29,33]. Recently, pelagic TTX-bearing larvae of flatworm *Planocera multitentaculata* have been proposed as the biological TTX source for Japanese Akazara scallops *Chlamys farreri* subsp. a*kazara*, based on the presence of *P. multitentaculata* DNA in the digestive gland, as well as the ability of mussels *Mytilus galloprovincialis* to acquire TTX through feeding on the larvae in a laboratory-based study [28]. *P. multitentaculata*, which generally contains high levels of TTX (up to 4000 mg/kg), is widely distributed in the Japanese Archipelago [34,35,36,37]; however, to our knowledge, this species has not been reported in Europe (WoRMS, MarBEF).

In addition to the flatworm genus *Planocera*, high TTX levels were found in ribbon worm *Cephalothrix simula* (Nemertea, Palaeonemertea) [38]. This species is native to the Northwest Pacific region and has been studied since the 1990s around the Japanese coast [38,39,40,41,42], where specimens with extreme toxicity up to 25,590 Mouse Units MU/g (~5120 mg/kg) were reported [38], and more recently in the Sea of Japan along the Far-Eastern Russian coast [43,44,45]. However, *C. simula* has increasingly been reported outside of its native environment: in Italy [46,47], the Mediterranean and Atlantic coasts of Spain [47,48], The Netherlands [49], France [47], and more recently in the UK [50,51]. The specimen collected from the southwest coast of the UK was the only *C. simula* from Europe that was subjected to toxin analysis. TTX and its analogues amounted to 54,300 µg/kg, while no TTX was found in other ribbon worm species (*Cephalotrhix rufifron* and *Tubulanus annulatus*) collected at the same time from the same location [51]. *C. simula* DNA was also confirmed at another coastal location in southern England during an eDNA study [50]. Coincidently, southern England has been identified as a higher-risk region for TTX occurrence in shellfish [20,24]*. C. simula* has the potential to be an important species in TTX trophic transfer, not least because it harbours high levels of TTX. Adult *C. simula* were preferentially consumed by TTX-bearing pufferfish *Takifugu niphobles)* during a laboratory study [52]; however, the presence of TTX in microscopic eggs and larvae [53,54] suggests that trophic transfer of TTX could be more complex and could involve filter-feeders. In this study, we explore the hypothesis that *C. simula* could be a potential source of TTX in British bivalves. Seawater and *M. gigas* samples were collected from a single location with a history of TTX in shellfish [11,20,24,27] and processed for molecular and chemical analyses to (1) determine the presence of *C. simula* in each of the monitored years, (2) investigate the genetic variability of the cytochrome c oxidase subunit *I* (CO*I*) of *C. simula* to design a qPCR assay able to detect all variants, (3) use the *C. simula* qPCR assay to determine the correlation between *C. simula* DNA abundance in seawater and TTX concentrations in *M. gigas*, and (4) apply the qPCR assay to investigate the presence of *C. simula* DNA in oyster digestive glands.

## 2. Results

### 2.1. Confirmation of C. simula in Seawater Samples

#### 2.1.1. Targeted Approach Using Novel *C. simula*-Specific Primers

Primers developed against a specific region of the mitochondrial CO*I* gene were used to screen eighteen seawater samples collected in the summers of 2019, 2020 and 2021 for *C. simula*. Successful PCR amplification was confirmed by gel electrophoresis against a positive control. Nine samples showing the most DNA amplification were subsequently sequenced using the Sanger method (Section 4.2.4). Trimmed consensus sequences (File S1) showed 100% identity to *C. simula*, e.g. GU807436 [52], and 97% and 93% sequence identity to the morphologically similar species *Cephalothrix mokievskii* MW118022 [55] and *Cephalothrix hongkongiensis* GU726613 [46].

#### 2.1.2. Intra-Species Variant Screening

To screen for CO*I* variants in the *C. simula* population within the study area, eighteen PCR products from the targeted assay were normalised by a dsDNA concentration and pooled and submitted for extensive Nanopore sequencing (Section 4.2.5). Consensus sequences generated using NGSpeciesID from the ~one million reads (947,894) showed that a single variant within the species was present. It was also noted that the sequences from this analysis (File S2) were identical to those from Sanger sequencing.

#### 2.1.3. Broad-Target CO*I* Amplicon Approach for Genus Variant Screening

A CO*I*-targeted amplicon approach was used to screen for diversity within the genus *Cephalothrix,* which was not possible using the *C. simula* species-specific assay. Two primer sets, developed by [56,57] (Section 4.2.6), were used to amplify metazoan CO*I* genes from a single seawater sample (15 June 2021), showing the greatest amplification in the targeted assay. DNA amplicons were submitted for Nanopore sequencing and aligned against the BOLD database. Folmer and Leray assays both generated reads aligning with the *Cephalothrix* reference, representing 0.2% and 0.7% of the total number of reads, respectively. Further alignment of consensus sequences indicated the presence of a single *C. simula* variant.

#### 2.1.4. Phylogenetic Analysis of Sequence Data

**Figure 1:**
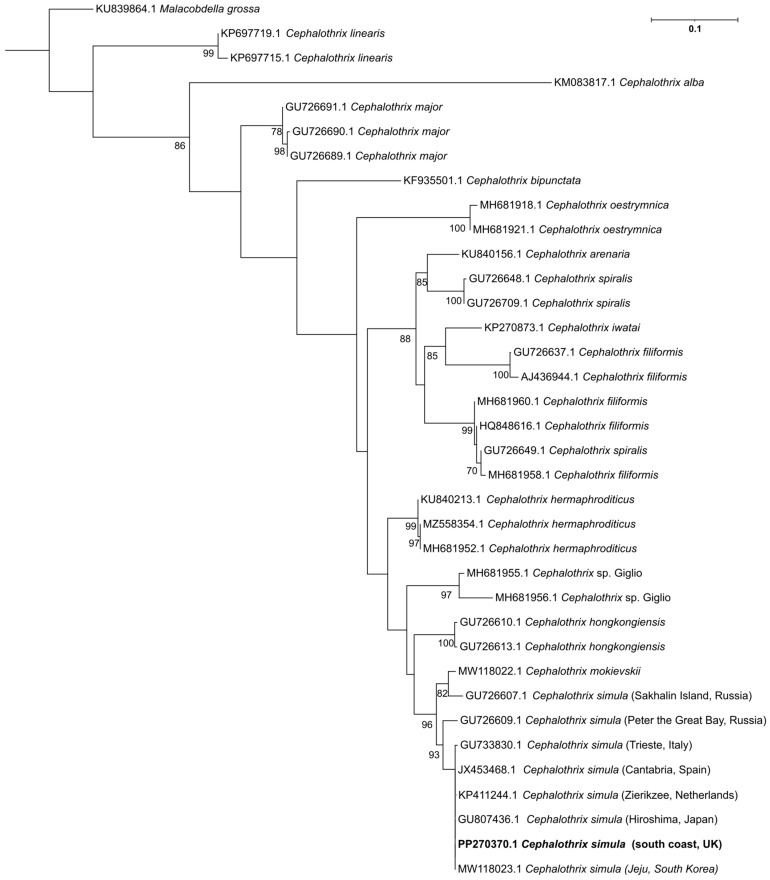
Maximum likelihood phylogenetic tree rooted with *Malacobdella grossa* using 10,000 bootstraps showing position of environmental sample characterised in this study (highlighted in bold) alongside *Cephalothrix* species (percent bootstrap values are shown on each node for values >80%).

The sequences generated by different primer sets (File S3) all fell within the CO*I* gene of the mitochondrial genome, with shorter amplicons also falling within the section amplified by the Folmer assay (Section 4.2.3). This enabled the alignment of sequences from each assay and showed that no variation was present. The data were positioned in a maximum likelihood phylogenetic tree populated with 35 different reference sequences, representing 14 species within the *Cephalothrix* genus (bootstrap value 10,000). The sequence generated from our study (PP270370) shared 100% identity with *C. simula* lineage, comprising specimens from the Northwest Pacific as well as from Europe: e.g., GU807436 from Hiroshima, Japan [52], MW118023 from Jeju Island, South Korea [55], GU733830 from Trieste, Italy [46], JX453468 from Cantabria, Spain [48], and KP411244 from Zierikzee, the Netherlands [49] (Figure 1). The next closest alignments were with specimens GU726607 and GU726609 from the Russian Far East coast (Sakhalin Island and Peter the Great Bay, respectively), which were originally identified as *C. simula* [46]; however, they were later assigned as “*Cephalothrix* sp. SCS-2010” and “*Cephalothrix* sp. 3 HC-2011”, respectively [58], or suggested to be a cryptic species *C. mokievskii* [59].

### 2.2. Abundance of C. simula DNA in Seawater and Comparison with TTX Concentrations in M. gigas

#### 2.2.1. Time Series Analysis

**Figure 2:**
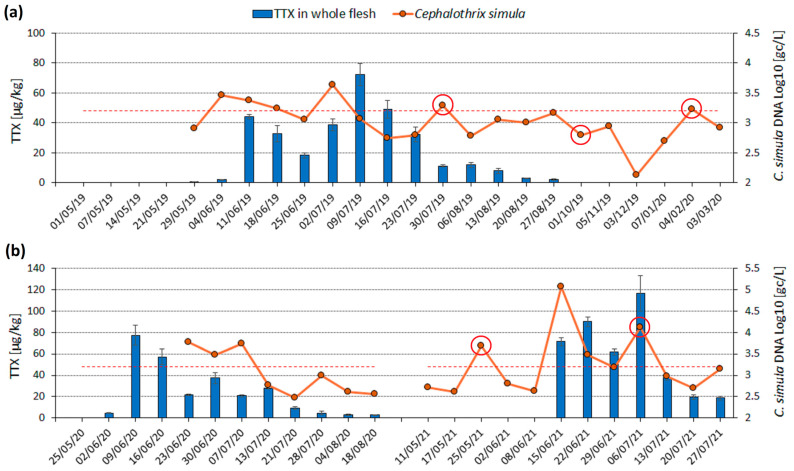
Time series of TTX concentration in *M. gigas* whole flesh (blue bars, primary *y*-axis) and *C. simula* DNA abundance in seawater (orange dots, Log_10_ values on a secondary *y*-axis) at a single location in (**a**) May 2019 to March 2020; (**b**) spring/summer 2020 and 2021. Red circles highlight the days with increased sediment content in seawater samples and red dashed lines show Log_10_ value of 3.2 (1600 genomic copies (gc)/L). TTX was measured in *M. gigas* on all dates specified on the date (x) axis, while *C. simula* DNA was measured on all days when seawater samples were taken (orange dots, n = 40). COVID-19 restrictions prevented seawater sampling in May and June 2020.

**Figure 3:**
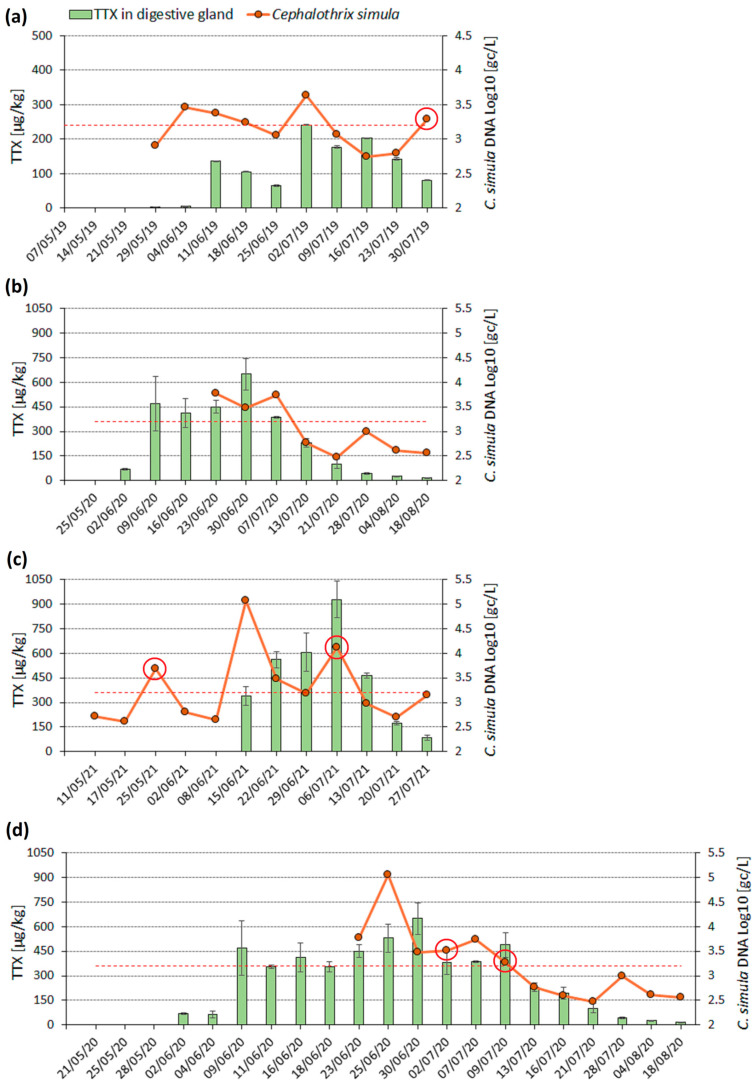
Time series of TTX concentration in *M. gigas* digestive glands (green bars, primary *y*-axis) and *C. simula* DNA abundance in seawater (orange dots, Log_10_ values on a secondary *y*-axis) at a single location in (**a**) 2019; (**b**) 2020 (**c**) 2021 and (**d**) 2020 during twice weekly sampling. Red circles highlight the days with increased sediment content in seawater samples and red dashed lines show Log_10_ value of 3.2 (1600 gc/L). TTX was measured in *M. gigas* on all dates specified on the date (x) axis, while *C. simula* DNA was measured on all days when seawater samples were taken (orange dots, n = 34). COVID-19 restrictions prevented seawater sampling in May and June 2020.

*C. simula* DNA abundance was measured at the location where TTX was found to accumulate in *M. gigas* annually. The parent TTX was monitored in whole flesh (Figure 2) for an extended time-series analysis and in the digestive glands during the higher-risk period for TTX accumulation (Figure 3). In 2019, a bimodal TTX distribution was observed in the whole flesh and digestive glands, with TTX maximum levels separated by four and three weeks, respectively (Figure 2a and Figure 3a). In 2020, the maximum TTX concentration in the whole flesh (77 µg/kg) was recorded on 9 June. Thereafter, the levels dropped by approximately 50% and ranged between 20 and 40 µg/kg from 23 June to 13 July (Figure 2b). More frequent (twice weekly) analysis of digestive gland samples in 2020 revealed TTX changes in more detail, with levels fluctuating between 350 µg/kg and 650 µg/kg over the entire month (9 June to 9 July), and the highest value recorded on 30 June (Figure 3d). In 2021, TTX in whole flesh resembled a bimodal distribution due to a 30% decrease in concentration on 29 June, whereas in the digestive glands, TTX continued to increase until 6 July (Figure 2b and Figure 3c). Although some differences in TTX patterns or in the timing of maximum TTX levels were observed between these two oyster matrices, their TTX concentrations were strongly correlated (r^2^ = 0.69, *p* < 0.001). Overall, TTX accumulation was dynamic and appeared to be seasonal; after the first occurrences in early June, TTX levels fluctuated for four to six weeks and generally did not follow a unimodal pattern. After the second week of July, TTX concentration dropped substantially, and no TTX was detected in *M. gigas* between October 2019 and March 2020 during the expanded monitoring (Figure 2a).

A novel *C. simula*-specific Real-Time qPCR was applied to seawater samples collected from the study area in 2019, 2020, and 2021, including at times outside of the main TTX accumulation period. All of the qPCR analyses had an amplification efficiency (as determined by the standard curves) between 92% and 99% (mean = 95%) and correlation coefficients of >0.99. The recovery of spiked *C. simula*-specific synthetic DNA was, on average, 92% ± 6%, indicating only a small level of inhibition in environmental DNA samples in qPCR assay.

The abundance of *C. simula* DNA in seawater varied between 10^2^ genomic copies per litre (gc/L) and 10^5^ gc/L and was visualised alongside TTX concentrations either in whole oyster flesh or digestive glands (Figure 2 and Figure 3). Generally, *C. simula* DNA levels below 1600 gc/L were recorded when *M. gigas* contained no TTX (prior 1st TTX peak in June) or after the last TTX peak in the second week of July. Inversely, levels above 1600 gc/L (Log_10_ = 3.2) were measured in June and July during the period of elevated TTX in *M. gigas*. More specifically, *C. simula* values above 2900 gc/L (Log_10_ = 3.46) on 4 June and 2 July 2019, 23 June–7 July 2020, 15 June, 22 June, and 6 July 2021 either coincided with spikes in TTX concentrations or preceded them by one week (Figure 2 and Figure 3).

However, in addition to the above observations, *C. simula* DNA exceeded 1600 gc/L outside of the main TTX accumulation period on three occasions. On these days (30 July 2019, 4 February 2020 and 25 May 2021), seawater samples were visibly turbid, suggesting an increased sediment content (Figure 2 and Figure 3). We did not attempt to measure *C. simula* DNA concentrations in sediment samples. While non-plankton life forms may persist, they are unlikely to be a food source for filter-feeding bivalves due to their larger size.

#### 2.2.2. Statistical Evaluation

**Figure 4:**
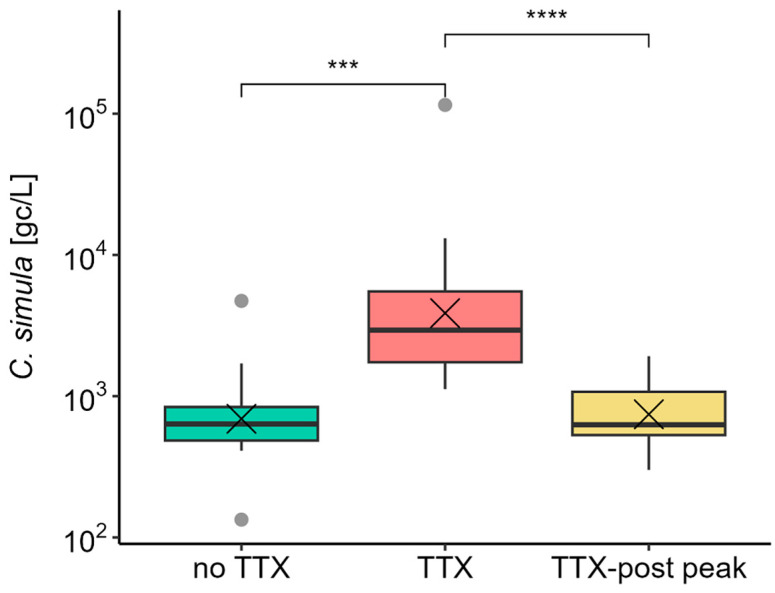
Box and whisker plot showing mean (cross), median (horizontal line), 1st and 3rd quartiles, outliers (grey dots) for *C. simula* DNA in seawater in each sample group. *** means significance *p* < 0.001, **** means significance *p* < 0.0001 (pairwise Wilcoxon test with “Bonferroni” adjustment of *p*-value).

To begin to evaluate the relationship between *C. simula* DNA abundance and TTX in *M. gigas* whole flesh, data from three sampling groups were compared (Table 1). Average *C. simula* DNA concentrations were approximately 10-fold higher (mean = 12,381 gc/L) in seawater samples collected during the active phase of TTX accumulation in *M. gigas* (“TTX” group) than in periods before or after (“no TTX” and “TTX-post peak” groups). Data values were Log_10_-transformed and found to be significantly different using the pairwise Wilcox test with “Bonferroni” multiple comparison adjustment (Figure 4). Non-normal distributions were confirmed visually using residual model analysis and the Shapiro–Wilk test (*p* = 0.0016). Excluding *C. simula* DNA concentrations measured in samples with high sediment content did not alter the overall outcome of the statistical analysis at the group level.

The relationship between *C. simula* DNA abundance in seawater and TTX in *M. gigas* whole flesh was further assessed using a Spearman’s rank correlation test. A moderate positive correlation was confirmed when five samples with increased sediment content were included (ρ = 0.48, *p* = 0.0016) and when these samples were excluded (ρ = 0.56, *p* = 0.0005). Correlation coefficients increased to 0.54 and 0.59, respectively, when TTX concentrations from digestive glands were applied instead of whole flesh data (Table 2).

**Figure 5:**
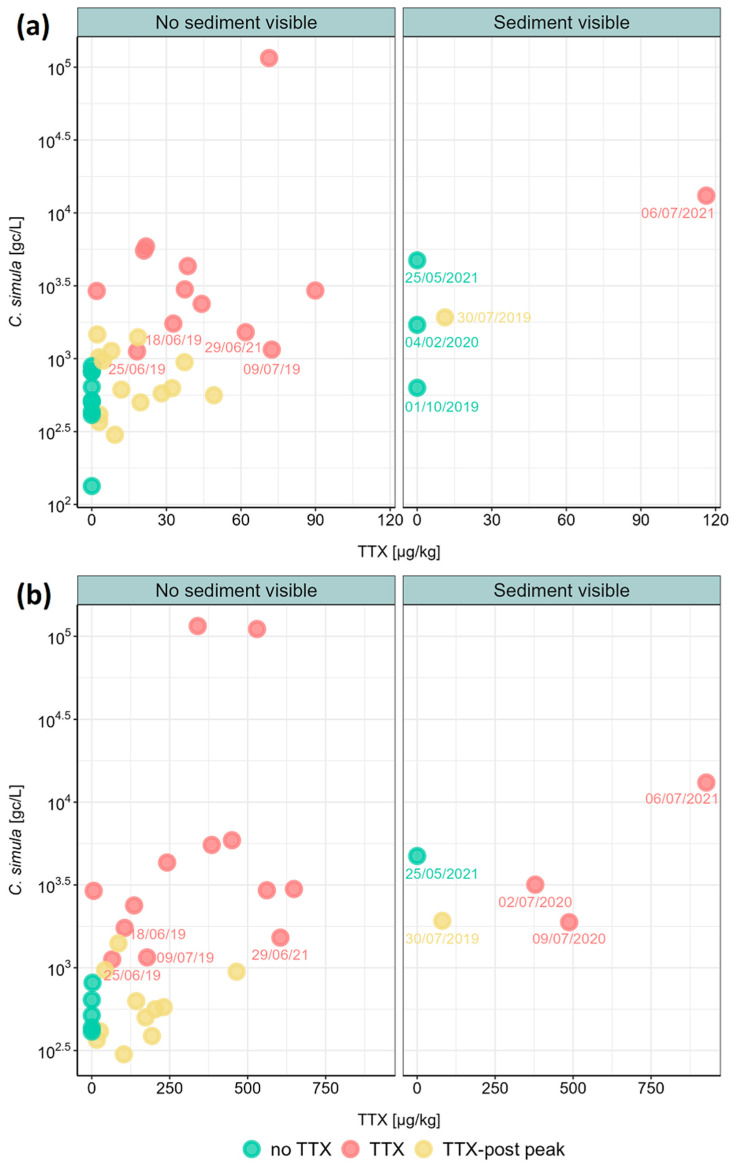
Concentrations of *C. simula* DNA in seawater and the corresponding TTX concentrations in *M. gigas* (**a**) whole flesh and (**b**) digestive glands. Individual scatter points represent mean concentrations of both variables and were colour-coded for each TTX group. Data points of a particular interest are highlighted with a label in DD/MM/YY format, including all data points when higher sediment content was observed in seawater samples.

By visualising TTX groups in a scatter plot, we could identify days when *C. simula* DNA values from the “TTX” group were comparable to values from the “no TTX” group (Figure 5a). We noted these corresponded to days either when TTX concentrations in *M. gigas* started to drop following its first peak (18 June 2019, 25 June 2019 and 29 June 2021) or to days of the last TTX peak in a particular year (9 July 2019), after which the toxin levels did not recover. Similar observations were made when TTX data from *M. gigas* digestive glands were applied instead of whole flesh (Figure 5b).

Information about all analysed seawater and shellfish samples, TTX and *C. simula* qPCR results, the presence of sediment, the volume of filtered seawater, and the allocation of samples to TTX groups is available in Table S1. An example of a chromatogram from a field sample containing TTX is shown in Figure S1.

### 2.3. C. simula Presence in Digestive Gland of TTX-Bearing M. gigas

*C. simula* DNA was detected in *M. gigas* digestive gland extracts from 15 June 2021, when the highest *C. simula* DNA abundance in seawater (115,361 gc/L) was recorded. This day also marked the start of the TTX accumulation period in 2021, when TTX concentration in the oyster digestive gland increased from “not detected” on 8 June to 340 µg/kg on 15 June. *C. simula* values in three independent digestive gland DNA extracts ranged from 0.35 gc/µL to 0.97 gc/µL, or 590 gc/g to 1617 gc/g digestive gland tissue (mean ± s.d 1057 ± 520 gc/g) (Table 3). In addition to *C. simula* presence in the tissue, it was also detected in digestive gland swabs taken from two different animals on 15 June 2021 (Table 3). Results from undiluted and diluted tissue and swab DNA extracts spiked with synthetic *C. simula* DNA did not indicate that the matrix interfered with the assay. All negative controls did not show any amplification.

## 3. Discussion

### 3.1. Confirmation of C. simula in the Study Area

Using molecular methods, we confirmed the presence of the TTX-bearing ribbon worm species *C. simula* in seawater samples from the study location in southern England. Phylogenetically, our samples clustered with specimens from Network 11, regarded as the *Cephalothrix* (=*Procephalothrix*) *simula* network [47]. The Network comprises individuals from the Northwest Pacific region [46,52], one individual from San Diego, USA [46], and European specimens from Italy [46], Spain [47,48], the Netherlands [49], and France, where they were initially assigned as *C. linearis* [47]. This broad distribution of *C. simula* might indicate an anthropogenic transfer of the species from the Northwest Pacific to Europe at some point in the past. Different routes of *C. simula* introduction have been suggested, including large-scale imports of *M. gigas* from Japan and British Colombia to France between the mid-1960s and mid-1970s and from France to the Adriatic lagoons of Italy in 1970 [52,60]. Such a scenario of introduction is plausible because *C. simula* was observed to frequently adhere to cultured *M. gigas* shells in Japan [38,39,40].

In the UK, *C. simula* was identified for the first time in 2018 at Godrevy Point in Cornwall [51]. Following this discovery, *C. simula* DNA was detected at another location on the southern coast [50], while more individuals were found at three more locations, also in southern England [61]. Together with the evidence of *C. simula* DNA in our study area, these combined reports are indicative of more established *C. simula* populations in this region than might have been initially thought. The route and timing of the introduction of this species are unclear, and besides accidental transport with *M. gigas* into Great Britain, including southern England [62,63,64], other transport routes such as ship-fouling or marine debris could be considered. Interestingly, while no commercial shellfish farming has been set up in the proximity of Godrevy Point, *Perophora japonica* and *Pikea californica*, two marine species native to the Pacific, were found at the same location as the *C. simula* specimen [51], suggesting that at least one introduction event has occurred at this site.

*C. simula* contains relatively high levels of TTX and TTX analogues that are thought to be important for prey immobilisation and/or for defence [42,65]. Thus far, all tested adult *C. simula* from the Northwest Pacific were found to be toxic, although the levels of TTX varied considerably between animals [38,39,40,43,44,45,66]. During an extensive survey involving over 764 individuals from different coastal habitats in Japan, toxicity ranged from approximately 33,800 µg/kg to 5120,000 µg/kg (calculated from reported values of 169 to 25,590 MU/g), while 48% of the specimens were classed as “extremely toxic”, containing TTX above 400,000 µg/kg (calculated from reported values of 2000 MU/g) [38]. In comparison, TTX and TTX analogues in *C. simula* from Cornwall, UK, amounted to 54,300 µg/kg [51]. To our knowledge, this was the only specimen from Europe subjected to TTX testing and reported in peer-reviewed literature.

### 3.2. Correlating C. simula DNA Abundance in Seawater and TTX Concentrations in M. gigas from the Study Area

After confirming *C. simula* DNA in our study area, a quantitative assay was applied to measure its abundance in seawater samples. The group analysis revealed significantly higher *C. simula* DNA abundance in seawater when TTX accumulated in *M. gigas* (“TTX” group), compared to periods of TTX absence in oysters (“no TTX”) or following the last TTX concentration peak (“TTX-post peak” group) (Table 1). Assigning data into the groups was problematic due to the non-unimodal distribution of TTX. This was particularly evident for days following the first TTX peak and before the secondary increase in TTX concentrations, e.g., on 18 June 2019, 25 June 2019, and 29 June 2021 (Figure 2). However, shifting these days from the “TTX” group to “TTX-post peak” did not change the overall outcome of the analysis.

The second approach revealed a moderate positive correlation between TTX in *M. gigas* whole flesh and *C. simula* DNA in seawater. The correlation coefficient increased after applying TTX data from the digestive glands, resulting in a borderline moderate/strong correlative relationship with *C. simula* (Table 2). Several factors might have impacted the strength of the correlation. Firstly, a daily rather than weekly analysis may better describe dynamic changes in the relatively short TTX accumulation period in *M. gigas*. To address this need, while considering logistics and available resources, the sampling frequency was increased from weekly to twice weekly in 2020. However, the early start of TTX season in 2020 was disrupted by COVID-19 restrictions, and therefore, water sampling before mid-June 2020 was not possible. In addition to a weekly collection, only four seawater samples were obtained and tested for *C. simula* after 23 June 2020 during the TTX accumulation period. Secondly, the qPCR assay did not distinguish between different life stages of *C. simula*, while only eggs and larvae up to a certain size could become a food source for *M. gigas* and, consequently, a source of TTX. Even in the absence of TTX in shellfish, *C. simula* DNA was measured at an average of 1019 gc/L (Table 1). We suspect that the “baseline” level in the water column would have increased only under two scenarios: during increased water turbidity triggered by windy conditions such as those observed prior to 25 May 2021 or during the spawning and formation of pelagic larvae. The limit of quantification was not calculated for the qPCR assay, and therefore, there is some uncertainty about the accuracy of lower concentrations of *C. simula* DNA. However, the sudden increase in *C. simula* DNA in seawater from approximately 640 gc/L and 434 gc/L on 2 and 8 June 2021, respectively, to 115,361 gc/L on 15 June 2021 could be explained by a spawning event. Despite the limitations outlined above, our results indicate that the accumulation of TTX in *M. gigas* could potentially be linked to an increased abundance of *C. simula* in the water column, as observed annually in late spring/early summer 2019–2021.

### 3.3. C. simula as a Potential Source of TTX in Bivalve Shellfish

The main aim of our study was to examine if *C. simula* could be involved in the trophic transfer of TTX to bivalve shellfish. The detection of *C. simula* DNA in oyster digestive gland samples is the strongest evidence that has been recovered thus far to support this hypothesis. Significantly, its presence coincided with the first day of TTX accumulation in 2021, and the day when the highest *C. simula* DNA concentrations were found in seawater. These changes were dynamic; TTX concentrations in digestive gland tissues increased from “not detected” on 8 June to 340 µg/kg seven days later, while *C. simula* in seawater increased 200-fold to 115,361 gc/L during the same time. However, despite large increases in seawater *C. simula* DNA, levels in digestive gland samples were very low and below the lowest level of calibration standard (1 gc/µL); similarly low levels were found from two of ten digestive gland swab samples taken on the same day. Recently, Biessy and colleagues failed to detect *Cephalothrix* sp. in the digestive glands of TTX-bearing *M. gigas* from Loperhet estuary, France, using droplet digital PCR (ddPCR) [25]. The very low to undetectable recovery of *Cephalothrix* sp. DNA in oysters at a time of peak TTX accumulation could suggest that *C. simula* was not a source of TTX, or it could indicate challenges with the detection methodologies. Uneven distributions of *C. simula* in the digestive tissue or rapid degradation of its DNA may hinder detection by molecular methods. Similarly, matrix effects, sensitivity, and specificity checks may not be fully explored due to a lack of reference material [25].

Associations between *C. simula* and *M. gigas* were first studied more than a decade ago: while hundreds of *C. simula* specimens were collected from *M. gigas* shells during an extensive survey in Japan, TTX-like toxic activity was not detected in oyster flesh from fouled shells using a bioassay [38]. Therefore, *C. simula* had not been initially considered as a TTX source for *M. gigas*, not at least through inhabiting/invading oyster shells. The digestion of an adult or juvenile metazoan ribbon worm would not be possible for filter-feeding bivalves, which source nutrients from microscopic particles (plankton, detritus, and bacteria), approximately between 5 and 200 µm in size [67,68,69]. However, recent findings of TTX-bearing flatworm larvae during TTX accumulation in Japanese Akazara scallops [28] and the confirmation of TTX presence in *C.* cf. *simula* eggs and larvae [53,54] have made the hypothesis that filter-feeding bivalves could acquire toxins from TTX-bearing invertebrates more plausible. The larval development and TTX content of *C.* cf. *simula* from Peter the Great Bay (Russian coast, Sea of Japan), according to authors belonging to the *C. simula* complex, was studied in laboratory conditions [53]. Each captured female released 10,000–15,000 spherical eggs, 50–80 µm in diameter, and the mean ± standard deviation of the TTX and total TTXs was 9.9 ± 1.8 ng and 12.2 ± 3.2 ng, respectively (n = 3) [53]. Spherical embryos hatched one day post fertilisation (dpf) and measured around 130 µm in diameter. In subsequent days, the pelagic larva elongated to an oval (4 dpf) and a teardrop shape (6 dpf), the latter measuring 290 µm in length and 100 µm wide at the anterior end. Despite being fed TTX-free food, the content of TTX and total TTXs in larvae did not significantly differ between development stages (1.5 dpf to 41 dpf, n = 5 per development stage) and varied between 6.2 and 8.5 ng TTX/larva, or 7.5–10.3 ng total TTXs/larva [53]. Assuming 100% uptake and no immediate depuration, approximately 300 larvae would need to be digested by a 20 g oyster to acquire 2000 ng of TTX, or 100 µg/kg whole flesh—the concentrations seen in our study. In addition to TTX content, information about TTX analogues is another important aspect to consider in trophic transfer and bioaccumulation/biotransformation research. Interestingly, TTX profiles in *C*. cf. *simula* larvae, consisting mainly of TTX (87%) and 5,6,11-trideoxy TTX (12%), remained similar to the profile in eggs throughout the observed development period (41 dpf), suggesting the retention of maternal TTXs [53]. TTX profiles in the whole *C.* cf. *simula* body seemed more complex; however, the proportion of TTX, 5,6,11-trideoxy TTX, and 5-deoxy TTX amounted to 93–98% of the total TTXs [44,45]. The *C. simula* specimen found in Cornwall also contained multiple TTX compounds, predominantly TTX (64%), followed by 6,11-dideoxy TTX (21%), 5,6,11-trideoxy-TTX (9%), and 11-oxo TTX (5%) [51]. Interestingly, the TTX profile in the whole flesh of *M. gigas* from our study was similar and consisted mainly of TTX (~ 69%), followed by 6,11-dideoxy TTX (~ 18%) and 5,6,11-trideoxy TTX (~ 13%). Utilising the same *M. gigas* samples from 2019, the changes in TTX and 6,11-dideoxy TTX proportions in whole flesh and digestive glands throughout the TTX accumulation period were reported previously [27]. The average proportion of 6,11-dideoxy TTX in the digestive gland was 27%, and it increased further to 36% on days with rising TTX levels. The lack of certified reference materials hindered the accurate measurement of TTX analogues, although estimated concentrations and qualitative assessments are valuable. In summary, our results presented in this paper, together with the new knowledge on the TTX content, profile, and size of *C.* cf. *simula* eggs and larvae [53], support the hypothesis that this ribbon worm species could be a potential TTX source for filter-feeding bivalves. Future research should include field studies on the distribution, spawning period, and TTX content of *C. simula* life forms, including pelagic larvae, from the same location as TTX-bearing bivalves. When comparing TTX profiles between organisms in a suspected trophic chain (e.g. between *C. simula* larvae and *M. gigas*), targeted TTX analyses by LC-MS/MS should be complemented with a non-targeted approach using high-resolution mass spectrometry (HRMS). Finally, controlled laboratory experiments are needed to confirm the ability of bivalves to bioaccumulate TTX through filter-feeding on *C. simula* eggs or larvae.

## 4. Materials and Methods

### 4.1. Sample Collection and Preparation

After obtaining the required permissions from relevant authorities, water and *M. gigas* samples were collected from a single trestle-farmed *M. gigas* production site with a history of TTX-positive shellfish [11,20]. The exact location of the sampling was anonymised to protect the identity of the shellfish grower. Samples were collected weekly between May and July/August in each of the three years: 2019, 2020, and 2021. In addition to weekly sampling in late spring and the summer months, monthly sampling was conducted between August 2019 and March 2020, and biweekly sampling was conducted between the end of May and mid-July 2020. No water samples could be collected between March and 22 June due to COVID-19 restrictions. However, during the period between 21 May and 22 June 2020, the shellfish producer was able to harvest and store shellfish in a freezer (<−15 °C), which were later collected and processed for TTX analysis.

Seawater was collected from a pontoon located approximately 400 m from the oyster trestles. To minimise variables associated with tides and to align water and shellfish sampling as closely as possible, seawater was taken around the time of low tide, which was acquired using tidal prediction software Poltips, version 3. Water was collected into sterile plastic containers using a sampling pole from a depth of approximately 0.5–1.0 m, without disturbing sediments, and transported in a chilled cool box to the laboratory. Upon arrival, one litre of water was filtered through a sterile 0.22 µm polyethersulfone (PES) membrane (Ø = 47 mm, Merck, Darmstadt, Germany) using a sterile filtration unit connected to a vacuum pump. An increased amount of sediment was noted in seawater samples on seven days (Table S1). The PES membrane was subsequently transferred into a 5 mL PowerWater DNA Bead Tube (DNeasy PowerWater kit, Qiagen, Hilden, Germany) using sterile forceps, without touching the filtered area, and stored at <−70 °C until DNA extraction (Section 4.2.1).

*M. gigas*, grown on trestles, were collected around low tide due to limited access at other tidal times. Live animals were transported to Cefas Weymouth Laboratory in a chilled cool box and kept at 10 ± 2 °C until processing (within 25 h after the collection). Animals collected between 21 May and 22 June 2020 could not be processed live due to COVID-19 restrictions. During autumn 2020, these oyster shells were taken out of the freezer and left to defrost at room temperature before being opened. In total, 20–25 animals were collected and split into two batches of 10–13 animals. Whole flesh from the first batch and digestive glands from the second batch were pooled and homogenised to obtain one representative sample per matrix on each sampling date. Digestive glands were dissected as described previously [27]. In 2021, an additional five animals were dissected on each sampling day, and an internal side of each digestive gland and stomach was swabbed by two flocked nylon 4N6FLOQSwabs (Thermo Fisher Scientific, Waltham, MA, USA), gaining ten swabs per sampling day. Samples were either stored in DNAase and RNAase-free cryovials at <−70 °C for DNA extraction (digestive gland tissue and swab samples, Section 4.2.2), or in 50 mL polypropylene tubes at < −15 °C for TTX chemical analysis (whole flesh and digestive gland samples, Section 4.3).

### 4.2. Molecular Methods

#### 4.2.1. DNA Extraction from Water Filters

PES filters (Section 4.1) were freeze-dried for ~1 h in an Alpha 2–4 freeze dryer (Martin Christ, Osterode am Harz, Germany) before DNA was extracted using DNeasy^®^ PowerWater^®^ Kit (Qiagen). A PW1 reagent, warmed at 55 °C, was added onto a filter in a PowerWater DNA bead tube, briefly mixed and incubated at 65 °C for 30 min to facilitate lysis, as recommended in the manufacturer’s protocol. The bead tubes were then homogenised at maximum speed for 5 min using a Vortex-Genie^®^ 2 mixer while being secured horizontally on a Vortex Adapter (Scientific Industries, Bohemia, NY, USA). The rest of the procedure was followed according to the manufacturer’s protocol, except for an additional (third) ethanol wash. To elute the DNA, 100 µL of EB solution, warmed at 70 °C, was added to the centre of the column and incubated at 70 °C for 5 min, then at room temperature for a further 5 min. DNA was eluted into a clean 1.5 mL microcentrifuge tube by centrifuging at 10,000× *g* for 1 min. DNA quantity and quality were checked using a NanoDrop 1000 Spectrophotometer (Thermo Fisher Scientific, Waltham, MA, USA). DNA was stored <−15 °C for short–term storage and <−70 °C for long-term storage. The summary of DNA extracted from water filters and subsequent molecular analyses is presented in Table S2.

#### 4.2.2. DNA Extraction from *M. gigas* Digestive Gland Tissue and Swab Samples

DNA from *M. gigas* digestive gland tissue and digestive gland swab samples were extracted using E.Z.N.A.^®^ Mollusc Kit (Omega Bio-Tek, Norcross, GA, USA). HiBind^®^ DNA Mini column and all the reagents were supplied with the kit, except molecular grade chloroform:isoamyl alcohol 24:1 (Merck, Darmstadt, Germany) and 100% molecular grade ethanol (Thermo Fisher Scientific, Waltham, MA, USA). Centrifugation was performed on MicroSTar 17R (VWR International Ltd, Radnor, PA, USA). Digestive gland homogenate aliquots were defrosted on ice, and 30 mg of each sample was weighed into a 1.5 mL microcentrifuge tube. Tissue samples were mixed with 350 µL of ML1 buffer and 25 µL of Proteinase K and incubated overnight at 37 °C. Defrosted digestive gland swabs were mixed with 450 µL of ML1 buffer and incubated at room temperature for 5 min. After further pulse vortex mixing, all buffer (~350 µL) was removed, and the same incubation process was followed as for tissue samples. The remainder of the extraction procedure was performed according to the manufacturer’s protocol (“Omega Bio-Tek E.Z.N.A.^®^ Mollusc DNA Kit, version 9.0”, 2020) with minor modifications in mixing and centrifugation times. After washing DNA trapped on the HiBind^®^ DNA Mini column, 50 µL or 75 µL of molecular grade UltraPureTM water (Invitrogen™, Thermo Fisher Scientific, Waltham, MA, USA) was added to the centre of the column and incubated at 70 °C for 5 min and at room temperature for further 5 min. DNA was eluted into a clean 1.5 mL microcentrifuge tube by centrifuging at 10,000× *g* for 1 min. DNA quantity and quality were checked and stored as detailed in Section 4.2.1. In total, 16 tissue extracts from 13 sampling days and 30 swab extracts from three sampling days (Table S1) were forwarded for *C. simula*-specific qPCR (Section 4.2.8).

#### 4.2.3. Targeted Approach Using Novel *C. simula*-Specific PCR Assay

**Figure 6:**
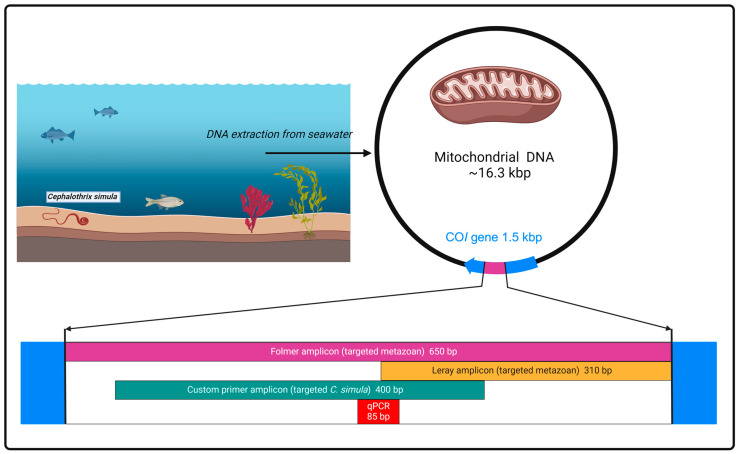
Graphical representation of four primer sets used in this study and relative position of their amplicons within CO*I* gene of mitochondrial plasmid DNA. Folmer and Mini CO*I* assays targeted all metazoan organisms, while custom assays targeted *Cephalothrix simula.* The figure was created with BioRender.com.

Novel primers specific to *C. simula* were designed by aligning 62 *Cephalothrix* reference sequences using BioEdit version 7.2.5 [70] (File S4). The reference sequences acquired from NCBI database included *C. simula* and other species within the genus. Forward (5′-GGKCAACCTGGTGCTTTAATAGG-3′) and reverse (5′-AAATGTATACCTCGTCATCGCA-3′) primers were designed to target ~400 bp region of the mitochondrial cytochrome c oxidase subunit *I* (CO*I*) gene (Figure 6).

Primer specificity was tested by BLAST analysis against the NCBI database [71]. A synthetic DNA strand from the same CO*I* region was used as a Positive Control Material (PCM). Nucleotide bases between primer binding sites were deliberately altered to be able to distinguish between environmental *C. simula* and synthetic PCM in confirmatory sequencing while maintaining amplification specificity (File S5-A). Primers and synthetic PCM were purchased from Invitrogen (Thermo Fisher Scientific, Waltham, MA, USA).

Each 25 µL PCR reaction contained 12.5 μL NEB Q5 Hot Start High Fidelity 2X Master Mix (New England Biolabs, Ipswich, MA, USA), 1.25 μL of each forward and reverse primer (final concentration 0.5 μM), 8.0 μL of molecular grade UltraPureTM water (Invitrogen, Thermo Fisher Scientific, Waltham, MA, USA) and 2.0 μL of undiluted DNA extract (Section 4.2.1). A negative control (molecular grade UltraPureTM water) and PCM diluted to approximately 1000 gc/μL in the background of 2 ng/μL Sheared salmon sperm DNA (Invitrogen, Thermo Fisher Scientific, Waltham, MA, USA) were also run in triplicate alongside samples. Thermocycling conditions on a Nexus X2 thermocycler (Eppendorf, Hamburg, Germany) were initial denaturation at 98 °C for 30 s, followed by 35 cycles of denaturation at 98 °C for 15 s, annealing at 64 °C for 15 s and extension at 72 °C for 30 s, final extension at 72 °C for 2 min and hold at 4 °C. For gel electrophoresis, triplicate PCR reactions were pooled, and 5 μL aliquots were visualised on 1.8% (*w/v*) agarose gel to check for satisfactory amplification in PCM and lack of amplification in negative control samples. The remaining pooled PCR reactions (60 µL per sample) were purified with NEBNext Sample Purification Beads (New England Biolabs, Ipswich, MA, USA) according to the clean-up protocol.

#### 4.2.4. Sanger Sequencing of Individual *C. simula* PCR Amplicons

Nine samples (Table S2) with the highest purified amplicon DNA concentration were sequenced unidirectionally by Eurofins Genomics TubeSeq Service (Eurofins Genomics, Ebersberg, Germany) using the forward Csim primer. DNA sequences for each sample were trimmed in MEGA software version 7.0.21 to remove primer sequences and any poor-quality data before being viewed in AliView software version 1.28 and run through BLAST against the nr database (accessed in January 2024).

#### 4.2.5. Intra-Species Variant Screening

To assess genetic variation in *C. simula* PCR products from the targeted assay (Section 4.2.3), eighteen water samples collected from the study area in summer 2019, 2020 and 2021 were sequenced using Nanopore technology (Table S2). Purified DNA amplicons were quantified using Qubit™ 1× dsDNA HS Assay with portable fluorometer Qubit 4 (Invitrogen, Thermo Fisher Scientific, USA) and diluted with molecular grade water to 5 ng/µL in each sample. The samples were then combined in equal proportions to obtain a single sample for sequencing on a XavION Mk1B device (Oxford Nanopore Technologies, Oxford, UK). Library preparation was completed using ligation sequencing kit LSK-110 in accordance with the Manufacturer’s protocol on a r9.4.1 flow cell (FLOW-MIN106, Oxford Nanopore Technologies, Oxford, UK). Base calling was performed using MinKNOW in super high accuracy mode. Sequencing was undertaken until over 1 million reads passing QC had been generated, at which point sequencing was stopped. Data were filtered so that only reads with a quality score greater than Q20 were used for onward bioinformatic analysis. Sequence reads were clustered using NGSpeciesID version 0.1.1.1 [72], while consensus sequences were generated by Spoa version 4.0.7, which were subsequently polished using Medaka version 1.2.4. Sequences with trimmed overhangs and primers were used to design *C. simula*-specific primers and probe for Real-Time qPCR (Section 4.2.8).

#### 4.2.6. Broad-Target CO*I* Amplicon Approach for Genus Variant Screening

To screen for additional species within *Cephalothrix* genus or potential variants of the *C. simula* that may not have been amplified by the targeted *C. simula* PCR assay, a broad-target CO*I* amplicon approach was undertaken. DNA extracted from a water sample on 15 June 2021, showing the strongest PCR band in the targeted assay, was used for PCR using two universal CO*I* primers protocols developed previously [56,57]. The Folmer assay used the forward primer LCO1490 (5′-GGTCAACAAATCATAAAGATATTGG-3′) and reverse primer HC02198 (5′-TAAACTTCAGGGTGACCAAAAAATCA-3′). The Leray assay used the forward primer mlCOIintF (5′-GGWACWGGWTGAACWGTWTAYCCYCC-3′) and the same reverse primer employed for the Folmer assay. The reaction composition for these two PCRs was the same as outlined in the publications with the following thermocycling conditions: Folmer assay consisted of 35 cycles at 95 °C for 60 s, annealing at 40 °C for 60 s, extension at 72 °C for 90 s, and a final extension step at 72 °C for seven minutes. The Leray assay used a “touchdown” PCR profile performing initial 16 cycles of denaturation at 95 °C for 10 s, annealing at 62 °C (−1 °C per cycle) for 30 s and extension at 72 °C for 60 s, followed by 25 cycles at annealing temperature 46 °C. PCR products were visualised using gel electrophoresis, cleaned up using magnetic beads and quantified by a Qubit 4 (Invitrogen, Thermo Fisher Scientific, Waltham, MA, USA). Samples were processed using the ligation sequencing kit LSK110 (Oxford Nanopore Technologies, Oxford, UK) in accordance with the manufacturer’s protocol. Nanopore sequencing was performed using the XavION custom sequencing platform [73]. Once sequencing was complete, data from each assay were filtered to remove low-quality reads (<Q9) with NanoFilt version 2.8.0, and MiniMap2 version 2.23-r1111 [74] were used to align reads against the CO*I* BOLD database [75] (public version accessed 23 September 2023). Reads aligning with *Cephalothrix* were extracted from the data and used to generate consensus sequences using NGSpceiesID with the process outlined in Section 4.2.5.

#### 4.2.7. Phylogenetic Analysis of Sequencing Data

Consensus sequences were oriented and aligned against 35 reference sequences from NCBI, comprising 14 species within the *Cephalothrix* genus. Primer regions and sequences with less than 20 supporting reads were removed. The aligned and trimmed sequences were then submitted for positioning into a phylogenetic tree using IQ-TREE with 10,000 bootstraps and using the maximum-likelihood model [76]. The resulting tree was visualised using the Interactive Tree of Life (ITOL) web tool (itol.embl.de).

#### 4.2.8. *C. simula*-Specific Real-Time qPCR

A novel Real-Time qPCR assay for the detection and quantification of *C. simula* was developed using the Primer3 software [77] to target 85 bp region of the consensus CO*I* gene sequences found in our samples. The primer sequences were as follows: forward primer Csim-F (5′-GGTGCTGTTGAAAGAGGTGT-3′), reverse primer Csim-R (5′-ATCTACAGAACCTCCAGCATG-3′) and fluorogenic Csim probe 5′-[FAM] CAGGATGAACTGTATATCCTCCT [QSY]-3′. Synthetic *C. simula* PCM (Invitrogen, Thermo Fisher Scientific, Waltham, MA, USA), representing the CO*I* region (File S5-B), was diluted to create a stock solution of 100,000 genomic copies (gc) per µL. The stock aliquots were stored at <−70 °C and used to prepare fresh calibration solutions (range 100,000 gc/µL–1 gc/µL) through ten-fold serial dilutions in 1× Tris-EDTA (Promega, Madison, WI, USA) in the background of 2 ng/µL sheared salmon sperm DNA for each assay to act as a standard curve. The 25 µL PCR reaction mix consisted of 1× Path-ID^TM^ qPCR Master Mix (Applied Biosystems, Waltham, MA, USA), 400 nM of each primer, 120 nM of Csim probe, 4.9 µL of molecular grade UltraPureTM water, 5 µL of *C. simula* PCM standard or 5 µL undiluted DNA extract. For a negative control, 5 µL of molecular grade UltraPureTM water was used instead of a DNA template. To check for PCR inhibition, all seawater and selected digestive gland DNA extracts were spiked with *C. simula* PCM at 50,000 gc/25 µL reaction and 500 gc/25 µL reaction, respectively. Each DNA extract was analysed in duplicate on a 96-well optical plate using QuantStudio^TM^ 3 system (Applied Biosystems, Waltham, MA, USA) with the following cycling conditions: an initial denaturation at 95 °C for 10 min, followed by 40 cycles of denaturation at 95 °C for 15 sec and annealing/extension at 60 °C for 1 min. The data were analysed in Design & Analysis Software version 2.6.0 (Thermo Fisher Scientific, Waltham, MA, USA). In total, DNA extracts from 44 seawater samples, 16 *M. gigas* digestive gland tissue samples and 30 digestive gland swab samples were analysed by *C. simula*-specific qPCR assay (Tables S1 and S2). Following qPCR, all amplification curves were visually inspected. Results were accepted if the amplification curves followed the typical sigmoidal shape and if the standard curves showed an efficiency of between 90% and 110% and had a correlation coefficient >0.98.

### 4.3. Analysis of Tetrodotoxins

#### 4.3.1. Reagents and Chemicals

Reagents for the sample extraction and subsequent Solid Phase Extraction (SPE) sample clean-up were HPLC grade or equivalent. Reagents for mobile phases and instrument washes such as water, Acetonitrile (MeCN), formic acid, and 25–31% ammonium hydroxide were all of LC-MS grade. TTX-certified reference material (CRM) (Cifga, Lugo, Spain) and non-certified TTX material (Enzo Life Sciences, Exeter, UK) were purchased to prepare TTX stock solutions. One Cifga TTX CRM ampoule (containing 25.9 ± 1.3 µg/g TTX) was opened, and the contents were diluted ten-fold with 0.25% acetic acid in deionised water and stored at <−15 °C. Enzo TTX powder was solubilised in 0.25% acetic acid to achieve a concentration of 10 µg/mL and stored at <−15 °C. Both stock solutions were further diluted with TTX-free *M. gigas* extract, which had been SPE-cleaned and diluted with MeCN in 1:3 ratio, to enable the preparation of matrix-matched TTX calibration solutions over six concentration levels (0.04–64.75 and 0.05–100 ng/mL for Cifga and Enzo solutions, respectively). The calibration solutions were kept in the refrigerated autosampler (<10 °C) and used within a week.

In the absence of certified control materials in the shellfish matrix, several samples have been utilised as TTX-positive controls for various stages of the TTX analysis. These include a non-certified mussel reference material (National Research Council Canada, Halifax, Canada), a Retention Time Marker (Cawthron Natural Compounds, Nelson, New Zealand) and a Laboratory Reference Material prepared in-house, all containing TTX and a range of TTX analogues as described before [27,78,79].

#### 4.3.2. TTX Extraction, Clean-Up and Dilution

For each sampling date, three 5.0 ± 0.1 g sub-samples and three 1.00 ± 0.01 g sub-samples were used to extract TTXs from *M. gigas* whole flesh and digestive gland, respectively. The exception was TTX analysis in digestive glands in 2019 when one 2.50 ± 0.01 g sub-sample per each sampling date was extracted and analysed in triplicate. A single dispersive extraction using 1% acetic acid was applied on homogenate samples, as previously validated [78,79]. The extracts were cleaned through Supelclean ENVI-Carb 250 mg/3 mL cartridges (Merck, Darmstadt, Germany) on an Aspec XL-4 SPE liquid handler (Gilson, Middleton, WI, USA) to remove some matrix interferences, mainly salts. The eluent was subsequently diluted with MeCN in 1:3 ratio into 700 µL Verex polypropylene autosampler vials (Phenomenex, Torrance, CA, USA). Samples were analysed by Liquid Chromatography–Tandem Mass Spectrometry (LC-MS/MS), and the concentrations were quantified using Cifga TTX matrix-matched calibration solutions.

To assess TTX recovery in whole flesh and digestive gland, samples collected from the same oyster farm and absent of detectable TTX were fortified with Enzo TTX stock solution at a concentration of 25 µg TTX/kg wet tissue. Three TTX-spiked whole flesh aliquots (5.0 ± 0.01 g), three TTX-spiked digestive gland aliquots (2.5 ± 0.01 g), and one non-spiked aliquot for each matrix were subjected to the same TTX extraction, clean-up and dilution procedure as environmental samples. Each extract was analysed by LC-MS/MS in triplicate in a single sequence, and the concentrations were quantified using Enzo TTX matrix-matched calibration solutions.

#### 4.3.3. Liquid Chromatography—Tandem Mass Spectrometry

An Agilent 1290 Ultra High-Performance Liquid Chromatography system coupled to an Agilent 6495B tandem quadrupole mass spectrometer (MS/MS) was used for TTX analysis (Agilent Technologies, Santa Clara, CA, USA). Since a Hydrophilic Interaction Liquid Chromatography (HILIC) column has been utilised (Acquity BEH Amide, 1.7 µm, 2.1 × 150 mm, in conjunction with a VanGuard BEH Amide guard cartridge, Waters, Milford, MA, USA), the analysis is often referred to as HILIC-MS/MS. The mobile phase composition, mobile phase gradients, injection volume, autosampler and column temperature settings were as described in previous work [51,78], with slight modifications of the gradient to suit the Agilent instrument. MS/MS conditions and the electrospray ionisation (ESI) interface were as follows: gas temperature 150 °C, gas flow 15 l/min, nebuliser gas 50 psi, sheath gas temperature 400 °C, sheath gas flow 12 l/min, Capillary voltage 2500 V. Multiple Reaction Monitoring (MRM) were acquired in positive ionisation mode and were taken from [51,78]. Primary (quantitative) and secondary (qualitative) MRM transitions for the parent TTX and TTX analogues were as follows: TTX and 4-epi-TTX (320.1 > 302.1, 162.1), 5,6,11-trideoxy TTX (272.1 > 254.1, 162.1); 11-nor TTX-6- ol (290.1 > 272.1, 162.1); 4,9-anhydro TTX (302.1 > 256.1, 162.1); 4,9-anhydro-5,6,11-trideoxy TTX (254.1 > 236.1, 162.1); 5-deoxy TTX (304.1 > 286.1, 176.1), 11-deoxy TTX (304.1 > 286.1, 176.1); 11-oxo TTX (336.1 > 318.1, 162.1); 4,9-anhydro-11-oxo-TTX (318.1 > 300.1, 162.1) and 6,11-dideoxy-TTX (288.1 > 270.1, 224.1). MRM transitions for arginine and hydroxy-arginine were also assessed in order to evidence effective chromatographic separation of TTX from these matrix co-extractives, which are known to affect TTX quantitative recovery and, therefore, method accuracy [78].

#### 4.3.4. Data Analysis

Results from the targeted TTX analysis by HILIC-MS/MS were processed using Agilent Technologies MassHunter Workstation software 10.2. Linear regression calibration curves using six-point matrix-matched standards with 1/X weighting were used to calculate the parent TTX concentrations (MRM transition 320.1 > 302.1, 162.1). Subsequently, TTX concentrations in the samples were adjusted for recovery as assessed in TTX-spiked experiments. LOQ for *M. gigas* whole flesh and digestive gland was 0.8 µg/kg and 2.5 µg/kg, respectively, based on values obtained from TTX-spiked samples. The presence of TTX analogues was carried out by comparing both MRM transition peaks and their associated ion ratios against those in TTX-positive controls (Section 4.3.1). Due to a lack of certified reference materials, TTX analogues were quantified against the TTX calibration curve, assuming an equimolar response. However, the estimated concentrations of TTX analogues were not combined with the parent TTX results and “total” TTX was not reported in this study.

#### 4.3.5. Statistical Analysis

Log_10_-transformed *C. simula* DNA values were used in all statistical analyses. For comparing *C. simula* between TTX groups, the normality of residuals was assessed by running a parametric test (ANOVA). Non-normality was suspected and then confirmed by the Shapiro–Wilk test; therefore, a non-parametric Kruskal–Wallis test was performed. To ascertain differences between individual TTX groups, a pairwise Wilcox test was applied, followed by Bonferroni correction of *p*-values. For correlative analysis between TTX concentration and *C. simula* DNA, the normality of residuals was assessed by running a linear model. Non-normality was observed; therefore, a non-parametric Spearman correlation test was applied. Statistical analyses were performed using R software Version 4.2.2 [80]. For all tests, *p*-values of < 0.05 were considered statistically significant.

## 5. Conclusions

The lack of knowledge about the biological source of TTX prevented a full understanding of the present and future risks posed by this toxin for shellfish consumers and the industry. In this extensive field study, the role of invasive TTX-bearing ribbon worm *C. simula* in TTX accumulation by bivalve shellfish was investigated for the first time. We were able to successfully confirm the presence of *C. simula* DNA in seawater at the location where TTX accumulated in *M. gigas* during the summers of 2019–2021. A statistically moderate correlation was found between *C. simula* DNA in seawater and TTX concentrations in *M. gigas*. More importantly, *C. simula* DNA was detected in oyster digestive glands from 15 June 2021, albeit at low levels. Although unequivocal evidence of *C. simula* as an immediate source of TTX is yet to be presented, this study has the potential to direct future efforts addressing the biological origin and trophic transfer of TTX in marine ecosystems. In addition, it highlights the potential negative impact of non-native invasive species on seafood safety.

## Figures and Tables

**Table 1:** Summary of TTX concentration in *M. gigas* whole flesh and *C. simula* DNA abundance in seawater at a single study area. Minimum, maximum and mean values from the three-year study (2019–2021) were calculated for each sample group. Allocation of samples into the groups was based on accumulation of TTX in *M. gigas*: “no TTX” = no TTX quantified, “TTX” = TTX quantified up to the last date of TTX increase, “TTX-post peak” = TTX quantified after the last TTX peak, after which concentrations have continuously decreased.

Sample Group (n = Number of Samples)	TTX Min–Max [μg/kg]	TTX Mean [μg/kg]	*C. simula* Min–Max [gc/L]	*C. simula* Mean [gc/L]
no TTX (n = 12)	0.0–<LOQ ^1^	0.0	134–4728 ^2^	1019
TTX (n = 13)	2.0–116.3	48.3	1122–115,361	12,381
TTX-post peak (n = 15)	2.2–49.1	16.1	300–1920	854

^1^ One sample collected on 29 May 2019 contained 0.6 μg/kg TTX, below Limit of Quantitation (LOQ) of 0.8 μg/kg. ^2^ One sample collected on 25 May 2021 contained 4728 gc/L by *C. simula* qPCR assay, when increased sediment content in seawater sample was also observed. The second highest *C. simula* concentration in this category was 1706 gc/L.

**Table 2:** Summary of Spearman correlation test results between *C. simula* DNA abundance in seawater and TTX concentrations either in *M. gigas* whole flesh (WF), or in digestive gland (DG), using all available data. The same test was repeated when five seawater samples with increased sediment content were excluded.

*C. simula* DNA Correlated with:	Correlation Coefficient (ρ)	Significance (p)	Degree of Freedom (n − 2)
TTX in WF (all data)	0.48	0.0016	38
TTX in WF (5 samples excluded) ^1^	0.56	0.0005	33
TTX in DG (all data)	0.54	0.0011	32
TTX in DG (5 samples excluded) ^1^	0.59	0.0008	27

^1^ seawater samples with increased sediment content were excluded.

**Table 3:** The summary of positive results from *C. simula*-specific Real-Time qPCR applied to *M. gigas* digestive gland samples. The table includes cycle threshold (Ct) values from the 1st and 2nd replicate analysis, the corresponding mean Ct values and *C. simula* concentration of genomic copies (gc) per µL of extract and per gram of digestive gland tissue. The main qPCR performance parameters were R^2^ = 0.999, slope = −3.42, efficiency = 96%. Ct values for calibration standards ranged from 19.19 in standard 1 (100,000 gc/µL) to 36.48 in standard 6 (1 gc/µL).

Sample Name (YYMMDD)	Ct			*C. simula* Mean	
	1st	2nd	Mean	gc/µL	gc/g
210615_Tissue Extract 1	36.79	37.44	37.11	0.58	965
210615_Tissue Extract 2	36.77	36.33	36.55	0.97	1617
210615_Tissue Extract 3	38.41	37.38	37.90	0.35	590
210615_Swab 4_oyster 2	38.42	38.58	38.50	0.22	na
210615_Swab 6_oyster 3	38.43	36.91	37.67	0.44	na

Ct = cycle threshold; na = not applicable.

## Data Availability

*C. simula* sequences generated in this study are deposited under GenBank accession number PP270370.

## Supplementary Materials

The following supporting information can be downloaded at https://www.mdpi.com/article/10.3390/md22100458/s1, Figure S1: Example of a chromatogram from a field sample containing TTX; File S1: Consensus *C. simula* sequences by Sanger method; File S2: Consensus *C. simula* sequences by Nanopore method; File S3: Consensus *C. simula* sequences from each assay by Nanopore method; File S4: *C. simula* primers design; File S5: Sequences of *C. simula* Positive Control Material (PCM); Table S1: Summary of results from chemical (TTX) and molecular (*C. simula*) analyses in *M. gigas* and seawater samples; Table S2: Summary of molecular analyses performed on seawater samples.
