# Supplementary material for: Investigating Non-Native Ribbon Worm Cephalothrix simula as a Potential Source of Tetrodotoxin in British Bivalve Shellfish
Source: Mar Drugs. 2024 Oct 5;22(10):458. doi: 10.3390/md22100458 (PMC11509275; doi:10.3390/md22100458)
Supplement: Supplementary file 1 [file marinedrugs-22-00458-s001.zip › Supplementary Figure S1.pdf]

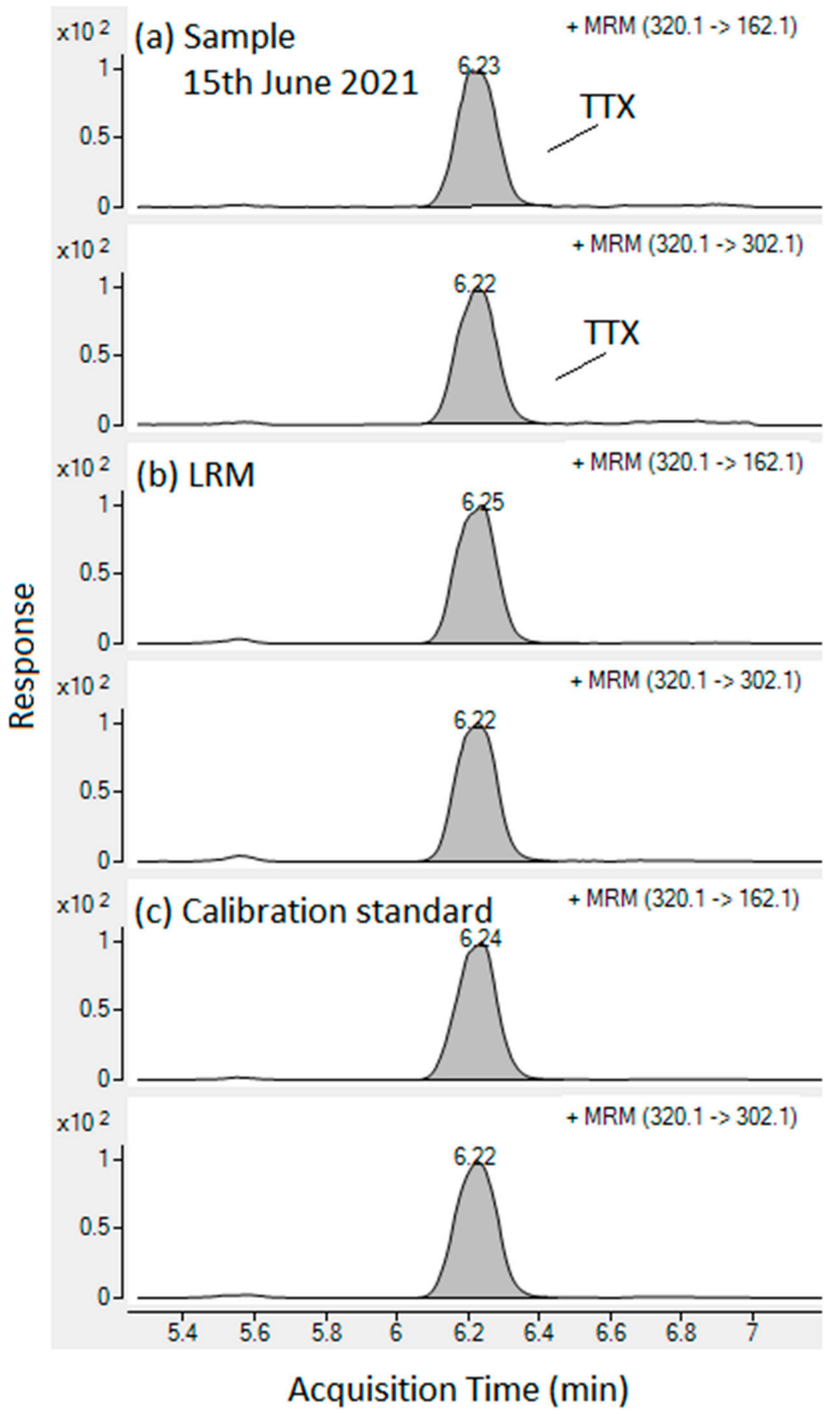

Supplementary Figure S1. TTX Multiple Reaction Monitoring (MRM) chromatograms in the *M. gigas* whole flesh field sample collected on 15th June 2021 (a) and in the positive control samples: (b) Laboratory Reference Material (LRM); (c) TTX calibration standard (5.18 ng/mL) prepared in the *M. gigas* whole flesh matrix.
