# Supplementary material for: Investigating Non-Native Ribbon Worm Cephalothrix simula as a Potential Source of Tetrodotoxin in British Bivalve Shellfish
Source: Mar Drugs. 2024 Oct 5;22(10):458. doi: 10.3390/md22100458 (PMC11509275; doi:10.3390/md22100458)
Supplement: Supplementary file 1 [file marinedrugs-22-00458-s001.zip › Supplementary File S5.pdf]

File S5-A: *Cephalothrix simula* synthetic positive control material used in conventional PCR and subsequent sequencing.

Bases in green represent forward primer biding site, bases in red represent reverse primer biding site and yellow bases were altered to distinguish synthetic PCM from environmental *C. simula* amplicon.

5’ TTAGGACAACCTGGTGCTTTAATAGGAGATGATCAATTGTATAATGTAATTGTTACTGCT

CATGCTTTTGTAATGATTTCCCTTTTGGTTATACTCGTAATAATTGGTGGTCTTGGAAAT

TGATTAGTTCCTTTAATATTAGGTGCTCCTGATATAGCTTTTCCTCGTATAGATAGTATA

AGTTCTTGATTGTTGCTCCCTGCTCTAACTCTACTTTTAGCTTCAGGTGCTGTTGAGAGA

GGTGTTGGAACAGGATGAACTGTATATCCTCCTCTATCAGGAAGTTTAGCTCATGCTGGA

GGTTCTGTAGATCTAGCTATTTCCTCTTTGCATTTAGCTGGAGTCTCTTCAATTTTAGGA

GCTATTAATCTTATTACAACTATTATTAATATGCGATGACGAGGTATACATTTTGAACGT

CTTC 3’

File S5-B: *Cephalothrix simula* synthetic positive control material used in Real-time qPCR

5’ TTAGGACAACCTGGTGCTTTAATAGGAGATGATCAATTGTATAATGTAATTGTTACTGCT

CATGCTTTTGTAATGATTTTTTTTTTTGGTTATACCCGTAATAATTGGTGGTTTTGGAAAT

TGATTAGTTCCTTTAATATTAGGTGCTCCTGATATAGCTTTTCCTCGTATAAATAATATA

AGTTTTTGATTGTTGCCCCCTGCTTTAACTCTACTTTTAGCTTCAGGTGCTGTTGAAAGA

GGTGTTGGAACAGGATGAACTGTATATCCTCCTTTATCAGGAAATTTAGCTCATGCTGGA

GGTTCTGTAGATTTAGCTATTTTTTCTTTGCATTTAGCTGGAGTTTCTTCAATTTTAGGA

GCTATTAATTTTATTACAACTATTATTAATATGCGATGACGAGGTATACATTTTGAACGT

CTTC 3’
