# Supplementary material for: Investigating Non-Native Ribbon Worm Cephalothrix simula as a Potential Source of Tetrodotoxin in British Bivalve Shellfish
Source: Mar Drugs. 2024 Oct 5;22(10):458. doi: 10.3390/md22100458 (PMC11509275; doi:10.3390/md22100458)
Supplement: Supplementary file 1 [file marinedrugs-22-00458-s001.zip › Supplementary Table S2.pdf]

Supplementary Table S2. Overview of water samples used for targeted and broad-target CO*I* amplicon molecular methods. The targeted approach utilised *C. simula*-specific primers while the broad-targeted approach utilised universal CO*I* gene primers. Samples were analysed individually, except for targeted Nanopore sequencing of *C. simula* DNA, where PCR products from eighteen samples in total were pooled together.

**Water samples**	***C. simula* PCR (targeted/individual)**	**Sanger sequencing (targeted/individual)**	**Nanopore sequencing (targeted/pooled)**	**Nanopore sequencing (universal CO*I* amplicon/individual)**	***C. simula* qPCR (targeted/individual)**
29th May 2019					✓
4th June 2019	✓	✓	✓		✓
11th June 2019	✓	✓	✓		✓
18th June 2019	✓		✓		✓
25th June 2019	✓		✓		✓
2nd July 2019	✓	✓	✓		✓
9th July 2019	✓		✓		✓
16th July 2019					✓
23rd July 2019					✓
30th July 2019					✓
6th Aug 2019					✓
13th Aug 2019					✓
20th Aug 2019					✓
27th Aug 2019					✓
1st Oct 2019					✓
5th Nov 2019					✓
3rd Dec 2019					✓
7th Jan 2020					✓
4th Feb 2020					✓
3rd March 2020					✓
23rd June 2020	✓	✓	✓		✓
25th June 2020[1]					✓
30th June 2020	✓	✓	✓		✓
2nd July 2020[1]					✓
7th July 2020	✓	✓	✓		✓
9th July 2020[1]					✓
16th July 2020[1]					✓

Water samples	*C. simula* PCR (targeted/individual)	Sanger sequencing (targeted/individual)	Nanopore sequencing (targeted/pooled)	Nanopore sequencing (universal CO*I* amplicon/individual)	*C. simula* qPCR (targeted/individual)
13th July 2020	✓		✓		✓
21st July 2020					✓
28th July 2020					✓
4th Aug 2020					✓
18th Aug 2020					✓
11th May 2021					✓
17th May 2021					✓
25th May 2021	✓		✓		✓
2nd June 2021	✓		✓		✓
8th June 2021	✓		✓		✓
15th June 2021	✓	✓	✓	✓	✓
22nd June 2021	✓	✓	✓		✓
29th June 2021	✓		✓		✓
6th July 2021	✓	✓	✓		✓
13th July 2021	✓		✓		✓
20th July 2021					✓
27th July 2021					✓
**Total**	**18**	**9**	**18**	**1**	**44**

[1] seawater samples collected in addition to weekly samples, associated with biweekly analysis of *M. gigas* digestive glands
